# Health Effects of Waterborne Contaminants: A Focus on Emerging Concerns

**DOI:** 10.3390/ijerph121012886

**Published:** 2015-10-15

**Authors:** Samuel Dorevitch

**Affiliations:** Division of Environmental and Occupational Health Sciences, School of Public Health, Institute for Environmental Science and Policy, University of Illinois at Chicago, Chicago, IL 60612, USA; E-Mail: sdorevit@uic.edu; Tel.: +1-(312)-355-3629

One of the Millennium Development Goals of the United Nations has been “To ensure environmental sustainability”, which includes the target of a 50% reduction by 2015 of “…the proportion of the population without sustainable access to safe drinking water and basic sanitation”. This year 147 countries have met the drinking water target, 95 countries have met the sanitation target, and 77 countries have met both [1]. While this is a major, positive development, to have readily available clean, safe water for drinking and recreation, however, remains beyond the reach of many people globally. Even in countries in which access to treated drinking water is widely available, new waterborne hazards continue to be identified and previously identified substances have been found to present newly-recognized threats to health.

This Special Issue of the *International Journal of Environmental Research and Public Health* addresses some of these emerging concerns. “Health Effects of Waterborne Contaminants: A Focus on Emerging Concerns” includes original research that characterizes specific waterborne health hazards in a lower-middle income economy [2], an upper-middle income economy [3], and high-income economies [4,5,6,7,8]. Other publications in this Issue address methods for risk assessment [9], regulation [10], and pathogen analyses [11], which are not specific to a given region or economic development category. The emerging concerns addressed include bacterial [2,4,5,8], viral [3], and protozoan pathogens [4,7], as well as the chemical hazards perchlorate [9] and microcystin [6]. Emerging concerns described in the Special Issue include those relevant to surface water [6,10], drinking water [2,4], water used in an occupational setting [5], water used in food production [3], or in multiple/unspecific settings [7,8,9].

The papers included in this Special Issue should be viewed not as a comprehensive review of emerging waterborne concerns, but rather as a step towards better recognition and characterization of these and other waterborne hazards. As these and other waterborne threats to public health are better understood, controls can be implemented as needed to keep populations healthy. It is with that larger goal of maintaining the health and well-being of populations by improving water quality, that this Special Issue has been published.
